# Detection of HTLV-1 replication and CD4+ proliferation in the humanized BLT mouse model

**DOI:** 10.1186/1742-4690-12-S1-O39

**Published:** 2015-08-28

**Authors:** Cynthia A Pise-Masison, Christopher C Nixon, Maria Fernanda de Castro Amarante, Robyn Washington Parks, Katherine McKinnon, Breanna Caruso, Veronica Galli, Steve Jacobson, Jerome Zack, Genoveffa Franchini

**Affiliations:** 1 Animal Models and Retroviral Vaccines Section, Vaccine Branch, NCI, Bethesda, MD, USA; 2 Department of Microbiology, Immunology and Molecular Genetics, University of California Los Angeles, Los Angeles, CA, USA; 3 Vaccine Branch Flow Cytometry Core, NCI, Bethesda, MD, USA; 4 Viral Immunology Section, Neuroimmunology Branch, NINDS, Bethesda, MD, USA

## 

Human T-lymphotropic virus type-1 (HTLV-1) is the causative agent of adult T-cell leukemia/lymphoma (ATL) and a number of lymphocyte-mediated inflammatory conditions including HTLV-1–associated myelopathy/tropical spastic paraparesis. Development of animal models to study the pathogenesis of HTLV-1–associated diseases has been problematic and the mechanisms driving disease remain poorly understood. Here we report our findings of HTLV-1 infection in humanized nonobese diabetic (NOD) severe combined immunodeficiency (SCID) common gamma chain knockout (NSG) mice that have been implanted with fetal bone marrow-liver-thymus tissue (BLT). BLT mice were injected with lethally irradiated CD4+ HTLV-1 producing cells. Blood samples were collected every four weeks to monitor CD4 and CD8 phenotypes and DNA isolation. Concomitant with an increase in the number of CD4+CD25+ T-lymphocytes in exposed animals compared to controls, we observed a significant increase in the HTLV-1 viral DNA load. Surprisingly, unlike what we observed in macaques, HTLV-1 virus mutated to knockout Orf-I protein expression was able to infect BLT mice and high viral DNA loads were detected. However, after sequencing the orf-I region of proviral DNA from infected animals, we found reversion of the point mutation to wild type. This was observed as early as four weeks after exposure, indicating in vivo viral replication and selection for Orf-I expression. Thus, the BLT mouse model successfully recapitulates HTLV-1 infection and may serve as an important tool for investigating in vivo mechanisms of HTLV-1 disease pathology and in evaluating drugs and treatment strategies.

